# Supplementary material for: A prefrontal cortex-lateral hypothalamus circuit controls stress-driven increased food intake
Source: Nat Commun. 2026 Mar 31;17:4620. doi: 10.1038/s41467-026-71073-z (PMC13199376; doi:10.1038/s41467-026-71073-z)
Supplement: Supplementary file 2 — Description of Additional Supplementary Files [file 41467_2026_71073_MOESM2_ESM.pdf]

Description of Additional Supplementary Files:

Supplementary Data 1: The statistical outcomes for the analyses performed for the datasets shown across main and supplementary figures.
